# Impact of the COVID‐19 Pandemic on School‐Based Medical Services in Austria

**DOI:** 10.1111/josh.70069

**Published:** 2025-08-26

**Authors:** Walter Hyll

**Affiliations:** ^1^ University for Continuing Education Krems (Danube University Krems) Krems an der Donau Austria

**Keywords:** COVID‐19, crisis management, pandemic, school health professionals, school health services

## Abstract

**Background:**

The COVID‐19 pandemic affected school students in several ways, including mental health issues, physical activity, or education. The objective of this paper is to shed light on an underexplored aspect of the pandemic by estimating its impact on the provision and utilization of routine school‐based medical services by school doctors in Austria.

**Methods:**

We utilize survey‐based data on school medical services collected by the Austrian Ministry of Education, Science and Research in the 2018/2019 and 2019/2020 school years. We compare both years using fixed‐effects regression.

**Results:**

In the 2019/20 school year, compared to 2018/19, we observe a decline in engagement of school health professionals in health‐promoting school projects (−4.3%‐points) and in first‐aid training sessions (−8.6%‐points). Most networking activities with other school‐based advisory teams decreased; only membership of a school‐based crisis team increased (+6.9%‐points). The share of compulsory check‐ups (−24.8%‐points) and student‐initiated medical consultations (−17.7%‐points) decreased significantly.

**Implications for School Health Policy, Practice, and Equity:**

Findings outline the need for strategies to provide school‐based medical services in crises, especially in countries where school health professionals are key points of contact for medical concerns.

**Conclusions:**

Our results indicate a significant reduction in routine school medical services during the pandemic.

## 1 Introduction

In many countries, school health services are an important pillar of public health. School health professionals provide a rich portfolio of tasks and activities [1]. Among these are screening students, offering advice to school management (e.g., school hygiene support), participating in parent‐teacher conferences and meetings, and giving lectures to teaching staff and students. In addition, school health professionals participate in meetings with faculty and the school community committee (SCC) and engage in (or even initiate) health‐related or health‐promoting school projects. In Austria, these services are provided by school doctors. In other countries such as the U.S., these services are often also provided by other medical professionals, such as school nurses.

Several studies demonstrate that health professionals in an educational setting are vital for identifying and improving students' health and play an essential role as a catalyst for accessing other relevant areas and professionals within the healthcare system [2, 3, 4, 5].

In March 2020, the Austrian government introduced measures to contain the transmission of the SARS‐CoV‐2 virus. The corresponding restriction of freedom of movement and employment nearly stopped all public and economic activities. Schools and kindergartens often closed their doors, cutting access to school‐based medical services. Delayed or omitted healthcare services can cause significant problems for an individual's health and a community's public health expenditures, specifically when the school health professionals are usually the first point of contact for a substantial number of children and young adults [6, 7, 8, 9, 10]. Against this background, the question arises as to what extent school doctors' range of routine services (i.e., their service catalog) changed in response to the COVID‐19 pandemic and how well children received (school‐based) medical care during this period. Understanding how healthcare utilization (or its absence) changed during the pandemic is vital for policy today.

Existing studies reveal that the COVID‐19 pandemic impacted school students in various ways. Research conducted globally highlights, for instance, that quarantine measures during the crisis had a detrimental effect on school children's mental health [11, 12, 13, 14, 15, 16]. Several authors emphasize the critical role of schools in addressing their students' mental health challenges [17, 18, 19].

The pandemic had also been linked to other adverse health outcomes. Due to the disruptions caused by COVID‐19, students were less physically active and exhibited unhealthy behaviors [9, 20, 21]. Consistent with this, recent studies report an increase in overweight and obesity rates, as well as a decline in the mental health and overall well‐being of children and adolescents [22, 23].

Moreover, COVID‐19 also severely disrupted education, halving the time students spent on school activities and leading to significant learning losses, with lower reading and math competencies, reduced schooling days, and potential long‐term income decreases of 3% [24, 25, 26, 27].

One aspect that has received less attention so far is the impact of the pandemic on school‐based medical services. Devore et al. [1] point out that “school physicians focus on the needs of individual children as well as the public health of the school community. They often assist schools in accommodating students who have special healthcare needs, manage acute and chronic illness, and oversee emergency response, environmental health and safety, health promotion, and education” (p. 178). That is, school‐based medical services play a non‐negligible role in students' health.

The retrieved literature provides insights into how school health professionals contributed to COVID‐19‐related issues and how their work practices changed. School nurses played a pivotal role in providing hygiene measures to avoid spreading the virus in schools or implementing new policies due to COVID‐19 [28]. Some qualitative studies address how school nurses adapted their work [29, 30]. School nurses' practices changed during the pandemic to deliver child‐focused services, shifting to virtual platforms for service delivery [31]. Nevertheless, evidence of the offered service portfolio and the services used is scarce. Some hints are provided by Sammut et al. [32] in a study based on an online survey of 78 participants. While 74% of the school nurses reported an increased workload during the COVID‐19 pandemic, about 60% experienced decreased contact with children, young people, or families. Furthermore, 86% reported that COVID‐19 restrictions impacted their ability to identify vulnerable children, young adults, and families and impaired adequate support. Another qualitative study based on interviews with 15 Maryland school districts also found that routine care was delayed during COVID‐19 [33]. A quantitative study shows that in Virginia, school‐based health services such as dental screening decreased due to the COVID‐19 pandemic [34]. The results are based on a cross‐sectional survey asking whether specific health services were provided before the pandemic and during the pandemic.

We contribute to the literature in several ways. We provide evidence of how routine services provided by school health professionals changed during the pandemic, complementing qualitative studies that cannot quantify the impact. Moreover, while previous studies focused on selected activities, such as screenings, our analysis examines a wide spectrum of services, offering a more comprehensive understanding of how the pandemic affected their overall performance.

## 2 Methods

### 2.1 Participants

The focus of the analysis is on federal schools such as Colleges for Higher Vocational Education or Academic Secondary Schools in Austria. Most of these schools are classified under International Standard Classification of Education (ISCED) levels 3–5 according to the 2011 ISCED and encompass school years 9–13, with some starting at year 5. Excluded from the analysis are Primary Schools (ISCED 1, years 1–4), Compulsory Secondary Schools (ISCED 2, years 5–8), the Pre‐vocational Schools (ISCED 3, year 9), and Part‐time Vocational Schools (ISCED 3, years 9–13).

In 2019, information on 445 federal schools with 271,556 students was collected (see Table S1). There were 570 doctors at these schools, 534 of whom took part in the survey. The response rate in 2019 was 82%. In 2020, 452 federal schools with 278,522 students participated in the survey. Of the 584 doctors responsible, 545 participated. We excluded schools from the dataset for which not all information was available. This affected schools for which several doctors were responsible, but not all of whom took part in the survey. In these cases, the information on activities at the school level was incomplete since doctors provided information only for their students and not for the entire school. Furthermore, we only considered schools that participated in both surveys, that is, the ones for 2019 and 2020.

Our final sample consists of 350 schools responsible for 213,766 students in 2019 and 215,309 students in 2020. In 2019, 411 doctors cared for the students, and in 2020, 412 doctors.

As robustness test, we further restricted the sample to schools with only one doctor, who had to be the same for the 2 years. This second subsample consists of 281 schools and about 149,000 students in each of the two school years. The number of doctors matched the number of schools, with 281 doctors for each year. Even though this additional restriction led to a reduction in the sample size, it allowed for controlling individual characteristics or properties of the doctors that did not change over the 2 years.

Based on observable school characteristics, such as the distribution of schools across federal states and school types, we tested whether the two analytical samples used in the analysis differed from the raw data. To do so, we conducted significance testing using chi‐square tests. Test statistics suggest that these distributions are not affected by a reduction in the sample size (see Table S1). This rough test at least does not indicate that there is a major selection bias in the subsamples.

### 2.2 Instrumentation

The data contains a wide range of activities. For the analysis, we subdivide the portfolio of activities of school doctors into five domains:

1. Participation in school committees (as part of advisory activities)
2. Health education
3. Networking activities with other school‐based advisory teams
4. Medical examinations
5. Medical consultations

Domain 1. Several advisory activities of school doctors take place within different school committees. This domain encompasses participation in parents' days and evenings, faculty meeting attendance, and meetings of the SCC. Since the possible survey responses to confirm that the respective activity had happened at least once a year were “No” or “Yes,” this information is considered as binary variables taking on the values 0 (for no) or 1 (for yes).

Domain 2. The second domain refers to health‐educational activities, including training the teaching staff and students in relevant subjects, contributing to health‐related or health‐promoting school‐based projects, and offering in‐school first‐aid training sessions. These educational activities are also coded as binary dummy variables.

Domain 3. We used survey questions on the prevalence of any interaction with school psychologists, youth coaches, school‐based social workers, and occupational health physicians to examine the networking activities of an Austrian school doctor with other school‐based advisory teams. Supplementing information relates to the membership in a school‐internal crisis team or a psychosocial support network. As with the previous variables, this information is available only in the “No” and “Yes” forms, coded as 0 and 1, respectively.

Domain 4. Medical examinations include the annual compulsory check‐up, school‐based medical examinations due to probable substance abuse, examinations upon request of the school management, and examinations of students whose health status requires monitoring and ongoing medical attention, henceforth called *surveillance students*. Note that an annual check‐up by the school doctor is required by law (according to §66 SchUG). Among others, height and weight of the student are recorded. Corrections of impaired vision or following up on recommended dental procedures are supposed to be tracked by the school doctor.

In contrast to activity domains 1–3, the surveys report the numbers of occurrences for various types of medical examinations. Also, survey data include information on the number of (written) parental notifications. To analyze activities in the medical examinations' domain, we express the associated variables as ratios calculated as frequencies relative to the overall number of students. In order to differentiate the figures from domains one to three, which depict proportions, we multiply the ratio per 100 and express figures as percentages, which also correspond to the number of medical examinations per 100 students. However, these numbers should not be mistaken for the proportion of students who have undergone medical exams because some students might be examined more often than others (e.g., those with an underlying health condition).

Domain 5. We group medical consultations into categories according to the nature of the request. The corresponding categories in the consultation domain are, thus, an acute complaint or acute illness, an injury, a (predominantly) psychosocial problem, an issue related to sexuality and sex education, issues with the class community (e.g., bullying), and other reasons. In addition, a variable is created that reflects the total number of all consultations. Medical consultations are also reported as numbers and are included in the analysis as frequencies relative to student numbers at the school of concern. Along the lines of domain 4, figures reflect the number of consultations per 100 students.

Table 1 displays the underlying regression samples. A descriptive comparison between 2019 and 2020 already suggests that almost all activities of school doctors were performed to a lower extent in the pandemic year.

**TABLE 1:** Descriptive characteristics of regression samples for 2019 and 2020.

	Full sample				1 doctor sample			
	2019	2020		Ha: diff ! = 0	2019	2020		Ha: diff ! = 0
	Proportions		Delta	Pr(|*T*| > |*t*|)	Proportions		Delta	Pr(|*T*| > |*t*|)
Participation in school committees								
Parents' day	0.35	0.32	−0.03	0.380	0.35	0.33	−0.02	0.594
Parents' evening	0.4	0.37	−0.03	0.485	0.41	0.37	−0.04	0.388
Faculty meeting	0.67	0.67	0.00	0.873	0.67	0.67	0.00	1.000
SCC meeting	0.19	0.19	0.00	1.000	0.19	0.17	−0.02	0.662
Health education								
Training of teaching staff	0.39	0.41	0.02	0.590	0.4	0.42	0.02	0.732
Training of students	0.57	0.56	−0.01	0.704	0.56	0.57	0.01	0.734
Participation in health‐projects	0.57	0.53	−0.04	0.255	0.55	0.52	−0.03	0.447
First aid training	0.18	0.09	−0.09	0.001	0.19	0.1	−0.09	0.002
Networking activities								
Occupational health physician	0.25	0.21	−0.04	0.324	0.22	0.2	−0.02	0.535
Youth coach	0.5	0.44	−0.06	0.130	0.48	0.42	−0.06	0.150
School psychologist	0.69	0.71	0.02	0.622	0.64	0.68	0.04	0.424
School social worker	0.26	0.21	−0.05	0.109	0.23	0.19	−0.04	0.177
School crisis team	0.74	0.81	0.07	0.031	0.75	0.82	0.07	0.051
School psychosocial network	0.67	0.66	−0.01	0.936	0.69	0.69	0.00	0.928

	Ratio (number per 100 students)		Delta	Pr(|*T*| > |*t*|)	Ratio (number per 100 students)		Delta	Pr(|*T*| > |*t*|)
Medical examinations								
Compulsory check‐up	93.26	68.49	−24.77	0.000	93.98	69.22	−24.76	0.000
Substance abuse examinations	0.08	0.3	0.22	0.233	0.07	0.31	0.24	0.289
Examinations upon request	0.67	0.84	0.17	0.512	0.67	0.63	−0.04	0.855
Parental notifications	20.11	13.62	−6.49	0.000	19.21	13.09	−6.12	0.001
Surveillance students	4.17	3.83	−0.34	0.424	4.1	4.03	−0.07	0.883
Medical consultations								
Acute illness	33.8	23.69	−10.11	0.000	31.28	22.34	−8.94	0.000
Injury	13.48	8.75	−4.73	0.000	12.82	8.36	−4.46	0.000
Psychosocial problems	6.63	5.06	−1.57	0.008	6.87	5.32	−1.55	0.026
Sexuality and sex education	1.29	0.9	−0.39	0.017	1.32	0.93	−0.39	0.044
Difficulties in the class community	1.15	0.94	−0.21	0.102	1.06	0.96	−0.10	0.450
Other reasons	4.29	3.59	−0.70	0.298	4.31	3.92	−0.39	0.606
Sum of consultations	60.65	42.94	−17.71	0.000	57.66	41.83	−15.83	0.000
Sample size (*N*)	350	350			281	281		

Abbreviation: SCC, school community committee.

### 2.3 Procedure

For the analysis of school‐based medical services, we utilize data from the Austrian Ministry of Education, Science and Research (BMBWF). The data is based on two surveys covering the school years 2018/2019 (hereinafter referred to as the 2019 survey) and 2019/2020 (hereinafter referred to as the 2020 survey). Both surveys are official surveys on the work of school doctors and are based on the employment contract of federal school doctors. School doctors for federal schools are employed by the federal government. The surveys were conducted after the end of each school year. In Austria, a school year (divided into two terms) starts at the beginning of September and ends at the beginning of July. The school closures due to the first wave of the pandemic took place from mid‐March to mid‐May 2020 and therefore affected the second term of the school year 2019/20, which was included in the 2020 survey. The doctors completed a separate questionnaire for each school at which they worked, resulting in a school‐level data set. Each school and doctor has a unique (anonymized) identifier, allowing us to track specific doctor–school pairings across both years.

These school doctors consented to use their voluntary annual (school medical activity) reports to the Ministry. Permission to access the raw data used in our study is provided by the Austrian Ministry of Education, Science and Research. The data used in this study were fully anonymized before its use. All methods were carried out in accordance with relevant guidelines and regulations.

### 2.4 Data Analysis

We utilize the panel nature of our dataset to examine the repercussions of the COVID‐19 pandemic on the volume and diversity of the activity portfolio of an Austrian school doctor. Additionally, the COVID‐19 pandemic and accompanying school closures can be considered as an exogenous shock.

To be more specific, our data corresponds to a (two‐period) balanced panel where each observation provides information both prior to and during the pandemic, allowing us to identify within‐subject changes. Thus, we can rule out the possibility that results are based on differences between schools. For example, comparing schools consistently exhibiting high levels of school health activity in 2019 with schools consistently exhibiting low levels in 2020 would yield misleading results.

In our analysis, we estimate fixed‐effects linear models, incorporating both observable and unobservable school‐specific fixed effects while adjusting for unobserved confounding. In essence, this estimation strategy controls for time‐invariant school characteristics such as environment, school type, regional attributes, or catchment area. Note that in our sample, the time‐invariant assumption applies only within a short period, namely 2 years. All standard errors are robust and account for heteroskedasticity.

The same estimation method is applied to all dependent variables. For binary dependent variables, these models are classified as linear probability models. Regressions are performed using Stata version 17.

## 3 Results

### 3.1 Main Results

We present our results along the five domains introduced in Section 2. The first domain accounts for advisory activities in connection with the participation of a school doctor in school committees. The regression results summarized in part 1 of Table 2 suggest that the percentage of schools where doctors participated in at least one parents' evening, one parents' day, and one faculty meeting declined in the school year affected by the pandemic. However, only the decrease in participation at a parents' day (−3%‐points) is statistically significant.

**TABLE 2:** Association between the pandemic year and advisory activities.

	1. Participation in school committees			
	(1)	(2)	(3)	(4)
Independent variables	Parents' day	Parents' evening	Faculty meeting	SCC meeting
Pandemic year	−0.031*	−0.026	−0.006	0.000
	(0.018)	(0.018)	(0.025)	(0.017)
School fixed effects	YES	YES	YES	YES
Constant	0.354***	0.397***	0.671***	0.189***
	(0.009)	(0.009)	(0.013)	(0.008)
*N*	700	700	700	700

	2. Health education			
	(1)	(2)	(3)	(4)
Independent variables	Training teaching staff	Training students	Health projects	First‐aid training
Pandemic year	0.020	−0.014	−0.043*	−0.086***
	(0.026)	(0.027)	(0.025)	(0.019)
School fixed effects	YES	YES	YES	YES
Constant	0.394***	0.571***	0.571***	0.180***
	(0.013)	(0.014)	(0.012)	(0.010)
*N*	700	700	700	700

*Note*: Regression coefficients are reported. Robust standard errors are provided in parentheses.

Abbreviation: SCC, school community committee.

*p < 0.1, **p < 0.05, ***p < 0.01.

Table 2 (part 1) also provides information on the importance of activities before the pandemic, captured by the constant. While at 67% of the schools a school doctor participated in (at least one) faculty meeting, the proportion of schools where a school doctor participated in a parents' day was 35%. With a participation rate of 19%, SCC meetings appear to be the least attractive activity for school doctors.

Part 2 in Table 2 depicts results for activities related to health education. We observe a declining engagement of school doctors in health‐related or health‐promoting school projects (−4%‐points) and (statistically insignificant) fewer training activities for students. Likewise, providing in‐school first‐aid training sessions also decreased significantly from 2019 to 2020: Whereas in 2019, in around 18% of the schools at least one first‐aid training course was offered by school doctors, in the pandemic year, the share was only 9%. Only the training activities for the teaching staff show an increasing, yet statistically insignificant, tendency.

Finally, Table 2 (part 2) reveals that in 2019, in a higher proportion of schools, doctors gave one or more educational talks to students (about 57%) than to teaching staff (about 39%). Also, in more than half of all schools, doctors were engaged in health‐related or health‐promoting school projects.

Another domain of school doctors' activities relates to contacting other school‐based advisory teams. Before the pandemic, in around two‐thirds of all schools, doctors contacted a school psychologist at least once a year or were part of a crisis team or psychosocial support network (see Table 3). Contacts with occupational health physicians or school‐based social workers were less frequent; only in one out of four schools did doctors contact them once a year or more often.

**TABLE 3:** Association between the pandemic year and network activities.

	Networking activities with other school‐based advisory teams					
(1)	(2)	(3)	(4)	(5)	(6)
Independent variables	Occupational Health doctor	Youth coach	School psychologist	School social worker	School crisis team	School psychosocial network
Pandemic year	−0.031	−0.057**	0.017	−0.051**	0.069***	−0.003
	(0.019)	(0.023)	(0.024)	(0.022)	(0.019)	(0.019)
School fixed effects	YES	YES	YES	YES	YES	YES
Constant	0.246***	0.497***	0.689***	0.260***	0.737***	0.666***
	(0.010)	(0.012)	(0.012)	(0.011)	(0.009)	(0.009)
*N*	700	700	700	700	700	700

*Note*: Regression coefficients are reported. Robust standard errors in parentheses.

*p < 0.1, **p < 0.05, ***p < 0.01.

Comparing prior‐ and post‐pandemic activities shows a few remarkable changes (see Table 3). On the one hand, we find that in fewer schools, doctors contacted occupational health physicians (statistically insignificant), youth coaches (significant) and school‐based social workers (significant) in the school year 2019/20 than in the year before. On the other hand, the proportion of schools with doctors who were part of a school‐based crisis team increased significantly by even seven percentage points. This increase might be directly related to the requirements imposed on students by the COVID‐19 pandemic. Interestingly, we observe neither an increase in contacts with school psychologists nor a greater participation in a psychosocial support network. Given the sharp increase in mental health problems [11, 12, 13, 14], one could also expect an increase in the related network activities.

The following domain of activities focuses on medical examinations and parental notifications (see part 1 of Table 4). Parental notifications represent an interesting metric; they directly affect a student's private environment and link the school doctor's work to their health status. As pointed out in Section 2, Table 4 reports all activities as numbers relative to the total number of students. The first regression in part 1 of Table 4 shows that the annual compulsory check‐up is of utmost importance for the Austrian school‐based medical service. Before the crisis, the number of examinations as a share of student numbers was 93%. We infer that even before the COVID‐19 pandemic jeopardized healthcare systems worldwide, some students fell through the cracks—with COVID‐19 worsening the situation. That is, the share of compulsory check‐ups significantly decreased by 25 percentage points to about 70% in the first year of the pandemic. This observation is quite unsettling because Austrian Law requires every student to undergo these medical exams once a year. In 2020, only around two‐thirds of the students at Austria's federal schools complied with this obligation, suggesting that the Austrian school‐based medical service had no pandemic backup plan.

**TABLE 4:** Association between the pandemic year and medical examinations and consultations.

	1. Medical examinations				
	(1)	(2)	(3)	(4)	(5)
Independent variables	Compulsory check‐up	Substance abuse examinations	Requested examinations	Parental notifications	Surveillance students
Pandemic year	−24.768***	0.221	0.172	−6.498***	−0.338
	(1.035)	(0.185)	(0.265)	(0.774)	(0.265)
School fixed effects	YES	YES	YES	YES	YES
Constant	93.261***	0.080	0.667***	20.113***	4.171***
	(0.518)	(0.093)	(0.132)	(0.387)	(0.132)
*N*	700	700	700	700	700

	2. Medical consultations						
	(1)	(2)	(3)	(4)	(5)	(6)	(7)
Independent variables	Acute illness	Injury	Psychosocial problem	Sexuality and sex education	Class community issues	Other reasons	All consultations
Pandemic year	−10.108***	−4.733***	−1.569***	−0.393***	−0.202**	−0.699	−17.705***
	(0.917)	(0.461)	(0.294)	(0.093)	(0.089)	(0.591)	(1.383)
School fixed effects	YES	YES	YES	YES	YES	YES	YES
Constant	33.797***	13.483***	6.632***	1.293***	1.147***	4.293***	60.646***
	(0.458)	(0.231)	(0.147)	(0.047)	(0.045)	(0.296)	(0.692)
*N*	700	700	700	700	700	700	700

*Note*: Regression coefficients are reported. Robust standard errors in parentheses.

*p < 0.1, **p < 0.05, ***p < 0.01.

Parental notifications also show a (significant) decrease of 6.5 percentage points, starting from a value of 20% before the crisis. We find no statistically significant effects of the pandemic for examinations due to substance abuse, those upon the school management's request, and those conducted due to students' specific medical needs. However, these activities did not often appear in the service portfolio before 2020.

Finally, we focus on activities related to medical consultations of students (see part 2 of Table 4). In contrast to school‐based medical examinations (see part 1 of Table 4), medical consultations happen upon a student's request and help to better understand student needs. The results reveal a decrease in all types of consultations, which are all significant except for consultations due to other reasons. Like network activities regarding school psychologists or psychosocial support networks, consultations due to psychosocial problems decreased surprisingly. This provides a strong indication that the physicians could not meet the needs of the students. In the school year 2018/19, the sum of all consultations was about 60% of the student population. In the early phase of the pandemic, in the spring of 2020, we observe a decrease of 18 percentage points.

### 3.2 Robustness Tests

In our main regressions, the unit of observation was schools with one or more doctors. As a robustness test, we restrict the sample to schools with only one doctor, with no doctor rotation between years. This allows us to control not only for school fixed effects, but also for the doctor‐specific fixed effects, accounting for time‐invariant characteristics such as education, attitudes, and skills. The restricted sample comprises 281 schools and doctors. All results remain qualitatively and quantitatively the same (see Tables S2–S6), suggesting that observed changes reflect actual shifts in service provision rather than changes in school or doctor composition.

We further test for heterogeneity by school type, estimating interaction models between school type and the pandemic year for key outcomes: compulsory checkups, parental notifications, and consultations (see Table S7).

The reduction in compulsory checkups was statistically similar across school types. For parental notifications, reductions were slightly smaller in Secondary Business Schools and Higher Federal Technical Colleges. This likely reflects the lower baseline levels in 2019 in these school types, limiting the potential for large declines. For consultations, the decline was also less pronounced in schools with initially lower consultation levels (Colleges for Early Childhood Pedagogy and Social Pedagogy, Secondary Schools for Economic Professions, and the “Other” category).

Lastly, we examine whether the pandemic's impact varies by schools' pre‐pandemic engagement in medical services, using interaction models based on whether baseline participation exceeded the median (see Table S8). For medical checkups, the decline was similar regardless of prior engagement. However, schools with higher initial levels saw significantly steeper declines in parental notifications and consultations. This pattern persists when using the 75th percentile as a cutoff, suggesting that larger declines were driven by higher starting activity levels rather than differential responses to the pandemic.

In sum, the pandemic had a broadly negative effect across all schools, with larger absolute declines in services at previously more active schools.

## 4 Discussion

Our study demonstrates that federal government measures to contain the SARS‐CoV‐2 pandemic significantly altered the range of services provided by school health professionals, primarily due to school closures. Analysis of data from before and during the pandemic reveals a substantial decline in direct service activities. Specifically, health education efforts dropped sharply, impairing an essential component of health promotion for children and adolescents in the pandemic's first year. Reduced participation of school doctors in health‐related projects and a decline in first‐aid training contributed to this downturn.

The pandemic also adversely affected the frequency of school‐based health check‐ups. This observation aligns with findings by Kranz et al. [34], who reported a similar decline in health screening activities during the pandemic. Since their results are based on a cross‐sectional survey and only account for selected activities using binary yes‐no questions, we extend their approach by analyzing a broader spectrum of services, which are also measured by their frequency of execution. Additionally, we use data from two survey waves, allowing us to observe changes over time.

A concerning outcome is the potential for missed diagnoses in conditions that are usually detected during routine check‐ups, as our findings also indicate a decrease in parental notifications. This interruption may have had particularly adverse effects on students who rely on school health services and therefore face difficulties accessing timely care.

Despite increased psychological strain on students during the pandemic [11, 12, 13, 14], networking activities among Austrian school health professionals, aside from crisis team participation, did not increase. This is concerning given the heightened mental health needs reported globally during the pandemic [33]. The reduced likelihood of contact with school psychologists and psychosocial support networks highlights a significant gap in mental health services for students, particularly when school‐based health professionals could play a pivotal role in addressing these needs or serve as intermediary [17].

### 4.1 Implications for School Health Policy, Practice, and Equity

The findings emphasize the need for better policies and practices for school health services, particularly during crises. The COVID‐19 pandemic revealed gaps, including reduced access to health education, screenings, and mental health services, underscoring the need for resilience‐building strategies. To address these issues, the following policy recommendations are proposed:

1. Governments should incorporate health services into crisis planning, ensuring that students maintain access to health education, screenings, and mental health resources during disruptions.
2. Schools should establish robust networks that include school psychologists and counselors to provide adequate psychosocial support.
3. Alternative service delivery methods, like telemedicine and online consultations, should be formalized to ensure continuity when in‐person services are unavailable [31].
4. To avoid lapses in early detection of health issues, schools should adopt comprehensive data collection and reporting methods, allowing for timely adjustments to health service provision based on real‐time needs assessments.

These policy measures could help strengthen the role of school health professionals as essential providers of early health intervention and health promotion.

### 4.2 Limitations

Limitations of this study include the binary nature of responses for some activities, which precludes determining the full scope of service changes and their impacts on student health. Nonetheless, robust evidence indicates that routine examinations and core services significantly decreased in the latter half of the 2019/20 school year, influencing the annual average. Furthermore, some non‐significant effects on binary outcomes may be explained by the fact that pandemic‐related restrictions only began in mid‐March 2020, allowing certain events (e.g., parents' evenings) to take place before school closures were implemented.

Additionally, our study is based on data from only two survey waves (2018/19 and 2019/20), limiting our ability to analyze long‐term trends or explore the possibility of a quick recovery post‐pandemic. However, it should be noted that there is an unpublished report for the 2017/18 school year indicating that the values correspond to those observed in 2018/19.

## 5 Conclusions

Our findings indicate that the pandemic exposed significant challenges in maintaining essential health services in schools, particularly through the reduction of health education, routine screenings, and mental health services. These disruptions highlight the vulnerability of school health systems during crises and emphasize the importance of integrating school health services into crisis management planning. Addressing these challenges through targeted policies and the establishment of flexible, crisis‐resilient service delivery models could help ensure the continuity of critical health services in future crises, which is essential, especially when student health is influenced by school health services.

## Ethics Statement

Ethical approval for this study was waived by the Ethics Commission of the University for Continuing Education Krems due to the type of study in accordance with Austrian Law, the Principals of the Helsinki Declaration, and the EU General Data Protection Regulation.

## Conflicts of Interest

The author declares no conflicts of interest.

## Supporting information


**Data S1:** Supporting information.

## Data Availability

The dataset Annual School Medical Activity Reports was provided by the Austrian Federal Ministry of Education, Science and Research. Due to legal and privacy restrictions, the data are not publicly available. Access may be granted upon request to ministerium@bmbwf.gv.at.
