# Supplementary material for: Impact of the COVID‐19 Pandemic on School‐Based Medical Services in Austria
Source: J Sch Health. 2025 Aug 26;95(12):1026–35. doi: 10.1111/josh.70069 (PMC12621155; doi:10.1111/josh.70069)
Supplement: Supplementary file 1 — Data S1: Supporting information. [file JOSH-95-1026-s001.docx]

**Supplementary Tables**

**TABLE S1**. Sample description

|  | **Raw sample** | | **Full sample** | | **1 doctor sample** | |
| --- | --- | --- | --- | --- | --- | --- |
| **Year** | **2019** | **2020** | **2019** | **2020** | **2019** | **2020** |
| Students per school (average) | 610.24 | 616.2 | 615.17 | 610.76 | 528.94 | 529.05 |
| Students (sum) | 271,556 | 278,522 | 213,766 | 215,309 | 148,632 | 148,663 |
| Doctors per school (average) | 1.28 | 1.29 | 1.17 | 1.17 | 1 | 1 |
| Doctors (sum) | 570 | 584 | 408 | 410 | 281 | 281 |
| Doctors participating in survey (sum) | 534 | 545 | 411 | 412 | 281 | 281 |
| Schools (sum) | 445 | 452 | 350 | 350 | 281 | 281 |
| **Federal state (percent)** | | | | | | |
| Burgenland | 4.3 | 4.2 | 4.9 | 4.9 | 4.3 | 4.3 |
| Carinthia | 6.3 | 8.2 | 5.7 | 5.7 | 6.4 | 6.4 |
| Lower Austria | 17.8 | 16.2 | 18.0 | 18.0 | 17.4 | 17.4 |
| Upper Austria | 18.0 | 17.7 | 19.1 | 19.1 | 19.9 | 19.9 |
| Salzburg | 6.7 | 7.3 | 7.4 | 7.4 | 7.8 | 7.8 |
| Styria | 15.3 | 15.5 | 10.3 | 10.3 | 7.8 | 7.8 |
| Tyrol | 8.1 | 8.0 | 8.9 | 8.9 | 10.3 | 10.3 |
| Voralberg | 3.6 | 3.5 | 4.3 | 4.3 | 5.0 | 5.0 |
| Vienna | 20.0 | 19.5 | 21.4 | 21.4 | 21.0 | 21.0 |
| Chi 2 Test compared to raw sample (p-value) |  |  | 0.8 | 0.5 | 0.3 | 0.2 |
| **School type (percent)** | | | | | | |
| Academic Secondary School | 50.3 | 52.2 | 52.0 | 52.0 | 49.5 | 49.5 |
| College for Early Childhood Pedagogy and Social Pedagogy | 3.1 | 3.1 | 3.1 | 3.1 | 3.9 | 3.9 |
| Secondary Business School/Secondary College of Business Administration | 18.7 | 17.9 | 19.1 | 19.1 | 23.1 | 23.1 |
| Secondary School for Economic Professions | 13.7 | 14.2 | 13.7 | 13.7 | 14.9 | 14.9 |
| Higher Federal Technical College | 11.7 | 10.6 | 11.1 | 11.1 | 7.5 | 7.5 |
| Other | 2.5 | 2.0 | 0.9 | 0.9 | 1.1 | 1.1 |
| Chi 2 Test compared to raw sample (p-value) |  |  | 0.7 | 0.9 | 0.2 | 0.3 |

Notes: Numbers for the subsamples are identical for the school years 2019 and 2020 since we only consider schools that participated in both survey years.

**Table S2.** Participation in school committees

|  | (1) | (2) | (3) | (4) |
| --- | --- | --- | --- | --- |
|  | **Parents’  day** | **Parents’ evening** | **Faculty meeting** | **SCC  meeting** |
| Pandemic year | -0.021 | -0.036* | 0.000 | -0.014 |
|  | (0.019) | (0.019) | (0.027) | (0.018) |
| School fixed effects | YES | YES | YES | YES |
| Doctor fixed effects | YES | YES | YES | YES |
| Constant | 0.352*** | 0.409*** | 0.665*** | 0.189*** |
|  | (0.009) | (0.010) | (0.014) | (0.009) |
| N | 562 | 562 | 562 | 562 |

Notes: Robust standard errors are provided in parentheses. SCC refers to school community committee ^*^ *p* < 0.1, ^**^ *p* < 0.05, ^***^ *p* < 0.01

**TABLE S3.** Activities related to health education

|  | (1) | (2) | (3) | (4) |
| --- | --- | --- | --- | --- |
|  | **Training teaching staff** | **Training students** | **Health  projects** | **First-aid training** |
| Pandemic year | 0.014 | 0.014 | -0.032 | -0.089*** |
|  | (0.029) | (0.031) | (0.026) | (0.022) |
| School fixed effects | YES | YES | YES | YES |
| Doctor fixed effects | YES | YES | YES | YES |
| Constant | 0.402*** | 0.559*** | 0.552*** | 0.185*** |
|  | (0.015) | (0.016) | (0.013) | (0.011) |
| N | 562 | 562 | 562 | 562 |

Notes: Robust standard errors in parentheses. ^*^ *p* < 0.1, ^**^ *p* < 0.05, ^***^ *p* < 0.01

**TABLE S4.** Network activities

|  | (1) | (2) | (3) | (4) | (5) | (6) |
| --- | --- | --- | --- | --- | --- | --- |
|  | **Occ. Health doctor** | **Youth coach** | **School psychologist** | **School social worker** | **School crisis team** | **School psy.soc. network** |
| Pandemic year | -0.021 | -0.060** | 0.032 | -0.046** | 0.068*** | 0.004 |
|  | (0.021) | (0.025) | (0.027) | (0.023) | (0.019) | (0.019) |
| School fixed effects | YES | YES | YES | YES | YES | YES |
| Doctor fixed effects | YES | YES | YES | YES | YES | YES |
| Constant | 0.221*** | 0.477*** | 0.644*** | 0.231*** | 0.751*** | 0.687*** |
|  | (0.010) | (0.012) | (0.014) | (0.011) | (0.010) | (0.009) |
| N | 562 | 562 | 562 | 562 | 562 | 562 |

Notes: Robust standard errors in parentheses. ^*^ *p* < 0.1, ^**^ *p* < 0.05, ^***^ *p* < 0.01

**TABLE S5.** Medical examinations and notifications

|  | (1) | (2) | (3) | (4) | (5) |
| --- | --- | --- | --- | --- | --- |
|  | **Compulsory check-up** | **Substance abuse exam.** | **Requested examinations** | **Parental notifications** | **Surveillance students** |
| Pandemic year | -24.760*** | 0.240 | -0.038 | -6.118*** | -0.070 |
|  | (1.131) | (0.226) | (0.211) | (0.791) | (0.306) |
| School fixed effects | YES | YES | YES | YES | YES |
| Doctor fixed effects | YES | YES | YES | YES | YES |
| Constant | 93.980*** | 0.069 | 0.666*** | 19.207*** | 4.100*** |
|  | (0.565) | (0.113) | (0.105) | (0.396) | (0.153) |
| N | 562 | 562 | 562 | 562 | 562 |

Notes: Robust standard errors in parentheses. Activities are presented as a proportion of the number of students. ^*^ *p* < 0.1, ^**^ *p* < 0.05, ^***^ *p* < 0.01

**TABLE S6.** Reasons for medical consultations

|  | (1) | (2) | (3) | (4) | (5) | (6) | (7) |
| --- | --- | --- | --- | --- | --- | --- | --- |
|  | **Acute illness** | **Injury** | **Psy.soc. problems** | **Sexuality and sex education** | **Class community issues** | **Other reasons** | **All consultations** |
| Pandemic year | -8.936*** | -4.458*** | -1.548*** | -0.390*** | -0.100 | -0.395 | -15.828*** |
|  | (0.941) | (0.477) | (0.344) | (0.107) | (0.089) | (0.659) | (1.426) |
| School fixed effects | YES | YES | YES | YES | YES | YES | YES |
| Doctor fixed effects | YES | YES | YES | YES | YES | YES | YES |
| Constant | 31.275*** | 12.822*** | 6.868*** | 1.321*** | 1.056*** | 4.314*** | 57.657*** |
|  | (0.471) | (0.238) | (0.172) | (0.053) | (0.044) | (0.330) | (0.713) |
| N | 562 | 562 | 562 | 562 | 562 | 562 | 562 |

Notes: Robust standard errors in parentheses. Activities are presented as a proportion of the number of students. ^*^ *p* < 0.1, ^**^ *p* < 0.05, ^***^ *p* < 0.01

**TABLE S7.** Heterogeneity of effects by school type

|  | (1) | (2) | (3) |
| --- | --- | --- | --- |
|  | **Compulsory check-up** | **Parental notifications** | **All consultations** |
| Pandemic year | -25.549^***^ | -7.737^***^ | -20.474^***^ |
|  | (1.401) | (1.211) | (1.902) |
| CECPaSP | -4.874 | -3.537 | -27.160^**^ |
|  | (6.797) | (9.040) | (11.945) |
| SBS/SCBA | -5.724^**^ | -7.579^**^ | -9.901 |
|  | (2.340) | (3.424) | (6.821) |
| SSEP | -3.219 | -4.864 | -19.815^***^ |
|  | (2.714) | (3.218) | (5.675) |
| HFTC | -5.931^**^ | -5.722 | -6.854 |
|  | (2.870) | (3.946) | (9.817) |
| Other | -18.107 | 11.705^***^ | -50.487^***^ |
|  | (18.438) | (3.280) | (5.505) |
| CECPaSP* Pandemic year | 2.395 | 1.307 | 15.386^***^ |
|  | (5.251) | (6.583) | (4.308) |
| SBS/SCBA*Pandemic year | 1.854 | 3.001^*^ | 3.545 |
|  | (3.275) | (1.704) | (4.345) |
| SSEP*Pandemic year | 3.263 | 1.009 | 7.732^**^ |
|  | (2.617) | (1.898) | (3.271) |
| HFTC*Pandemic year | -1.023 | 4.302^*^ | 2.716 |
|  | (2.986) | (2.566) | (4.388) |
| Other*Pandemic year | 2.013 | 0.631 | 28.489^***^ |
|  | (19.224) | (9.809) | (10.552) |
| Constant | 95.768^***^ | 22.880^***^ | 67.309^***^ |
|  | (0.979) | (2.256) | (3.551) |
| N | 700 | 700 | 700 |

Notes: OLS regression with interaction terms between school type and pandemic year. Clustered standard errors at the school level in parentheses.

Base school type: Academic Secondary School.

CECPaSP = College for Early Childhood Pedagogy and Social Pedagogy;

SBS/SCBA = Secondary Business School / Secondary College of Business Administration;

SSEP = Secondary School for Economic Professions;

HFTC = Higher Federal Technical College;

Other category consists only of three schools: one school for the deaf and two laboratory schools (university-affiliated institutions that integrate teacher training and pedagogical innovation).

^*^ *p* < 0.1, ^**^ *p* < 0.05, ^***^ *p* < 0.01.

**TABLE S8.** Heterogeneity of effects by school-level medical engagement

|  | (1) | (2) | (3) |
| --- | --- | --- | --- |
|  | **Compulsory check-up** | **Parental notifications** | **All consultations** |
| Pandemic year | -24.029^***^ | -0.111 | -5.017^***^ |
|  | (1.699) | (0.420) | (0.796) |
| High engagement | 14.764^***^ | 28.844^***^ | 67.534^***^ |
|  | (1.497) | (2.313) | (3.501) |
| High engagement*pandemic year | -1.487 | -12.774^***^ | -25.375^***^ |
|  | (2.068) | (1.391) | (2.413) |
| Constant | 85.921^***^ | 5.692^***^ | 26.879^***^ |
|  | (1.460) | (0.258) | (0.897) |
| N | 700 | 700 | 700 |

Notes: OLS regression with interaction between pandemic year and school-level engagement. Clustered standard errors at the school level in parentheses.

High engagement is defined as schools above the median in baseline participation for each outcome in 2019:

- Compulsory checkups ≥ 98.96%
- Parental notifications ≥ 12.31%
- Consultations ≥ 47.02%

Base engagement: Low engagement defined as below median.

p < 0.1, ** p < 0.05, *** p < 0.01.
